# Sequence Conservation and Functional Constraint on Intergenic Spacers in Reduced Genomes of the Obligate Symbiont *Buchnera*

**DOI:** 10.1371/journal.pgen.1002252

**Published:** 2011-09-01

**Authors:** Patrick H. Degnan, Howard Ochman, Nancy A. Moran

**Affiliations:** Department of Ecology and Evolutionary Biology, Yale University, New Haven, Connecticut, United States of America; Universidad de Sevilla, Spain

## Abstract

Analyses of genome reduction in obligate bacterial symbionts typically focus on the removal and retention of protein-coding regions, which are subject to ongoing inactivation and deletion. However, these same forces operate on intergenic spacers (IGSs) and affect their contents, maintenance, and rates of evolution. IGSs comprise both non-coding, non-functional regions, including decaying pseudogenes at varying stages of recognizability, as well as functional elements, such as genes for sRNAs and regulatory control elements. The genomes of *Buchnera* and other small genome symbionts display biased nucleotide compositions and high rates of sequence evolution and contain few recognizable regulatory elements. However, IGS lengths are highly correlated across divergent *Buchnera* genomes, suggesting the presence of functional elements. To identify functional regions within the IGSs, we sequenced two *Buchnera* genomes (from aphid species *Uroleucon ambrosiae* and *Acyrthosiphon kondoi*) and applied a phylogenetic footprinting approach to alignments of orthologous IGSs from a total of eight *Buchnera* genomes corresponding to six aphid species. Inclusion of these new genomes allowed comparative analyses at intermediate levels of divergence, enabling the detection of both conserved elements and previously unrecognized pseudogenes. Analyses of these genomes revealed that 232 of 336 IGS alignments over 50 nucleotides in length displayed substantial sequence conservation. Conserved alignment blocks within these IGSs encompassed 88 Shine-Dalgarno sequences, 55 transcriptional terminators, 5 Sigma-32 binding sites, and 12 novel small RNAs. Although pseudogene formation, and thus IGS formation, are ongoing processes in these genomes, a large proportion of intergenic spacers contain functional sequences.

## Author Summary

Endosymbiotic associations, such as that between *Buchnera aphidicola* and its aphid hosts, persist for millions of years and result in substantial changes to symbiont genomes. Most notably, symbionts exhibit reductions in genome size, the accumulation of slightly deleterious mutations, and rapid rates of evolution of gene sequences. Genomic studies have enabled the identification of the specific metabolic contributions of bacteria to their hosts; however, such reconstructions have had limited success recognizing the functional elements present in intergenic regions. Non-coding regions can result from reductive evolution and genome decay, *i.e.* through the inactivation of previously functional regions; but they may contain any of several classes of regulatory control elements that have been maintained over the history of the symbiosis. We produced complete sequences for two additional *Buchnera* genomes to identify stretches of intergenic DNA that have been conserved for more than 65 million years. Many of these regions contain identifiable elements involved in the regulation of transcription, and several are predicted to encode small non-coding RNAs, riboswitches, and new regulatory binding sites. Despite the continuous inactivation and erosion of sequences, and the loss of most ancestral regulatory mechanisms, a large portion of intergenic spacers in *Buchnera* maintains functional sequence elements.

## Introduction

Obligate bacterial symbionts possess the smallest cellular genomes [1]. Due to their extreme genome reduction, they retain only small fractions of ancestral gene sets. Comparisons of complete genome sequences within clades have revealed that genomes of obligate symbionts are stable, with few rearrangements and no uptake of novel genes over millions of years of evolution (e.g., [2]–[5]). The principal changes in these genomes are rapid sequence evolution combined with the ongoing erosion and loss of genes due to a mutational bias toward deletions [6]–[7]. This removal of nonfunctional sequences is a unidirectional process leading to ever-shrinking gene sets.

Protein-coding genes are relatively easy to recognize in these genomes, based on lengths of undisrupted open reading frames (ORFs) and on their clear homology to proteins encoded in other bacterial genomes. Functional inferences based on such homology have yielded insights into symbiont roles within hosts by establishing the specific metabolic capabilities that are retained in symbionts (e.g., [8]–[10]). In contrast, the intergenic spacers in these small genomes are more enigmatic. On account of the ongoing gene erosion and loss, many spacers consist, in whole or part, of inactivated pseudogenes in varying stages of decay [11]. At the same time, some sequences within intergenic spacers represent functional elements that are retained for their roles in gene regulation [12]. These are of particular interest because regulatory processes are the least understood aspects of symbiont genomes, which have lost most ancestral regulatory mechanisms [13]–[14].

Discriminating between decaying genes and functional elements within spacer regions is difficult in that inert sequences can be of varying sizes and base compositions and there is no single model for recognizing functional motifs. In *Buchnera aphidicola*, which has coevolved for over 150 million years with its aphid hosts, intergenic spacers occupy about 15% of the genome [15], which is within the typical range for bacterial genomes [6], [7]. These spacers contain a mixture of neutral sequences and functional elements, but the latter are largely unrecognized and undefined.

One way in which the functional relevance of a sequence can be assessed is through experimental disruption; however, obligate symbionts including *Buchnera* cannot be cultured in the laboratory, limiting most experimental approaches for linking sequences to functions. However, comparative genome analysis can reveal functional sequences in these genomes, since sequences under either purifying (negative) or positive selection will exhibit distinct patterns of evolution. But this approach relies on genomic sequences that are of appropriate levels of divergence in order to manifest meaningful signals in such comparisons. In the past, available genomes for *Buchnera* corresponded to four very distantly related aphid species that were too divergent for alignment of most intergenic spacers [16]. Conversely, multiple genomes available from *Buchnera* of a single host species, the pea aphid (*Acyrthosiphon pisum*), are nearly identical (<0.3% divergence), precluding the discrimination of sequences under different functional constraints [17].

In this study, we compare genomes of eight *Buchnera* of varying divergence times to monitor the origination and decay of intergenic spacers from previous protein-coding regions and to detect evolutionarily conserved elements within spacers. Two *Buchnera* genomes were newly sequenced and annotated for this study to permit analyses at evolutionarily relevant levels of sequence divergence. An advantage of *Buchnera* species for this phylogenetic footprinting approach is the complete conservation of gene order and orientation, allowing orthology of spacers to be assigned with confidence even when sequence divergence is high.

## Results

Of the eight *Buchnera* genomes analyzed, six were previously published, including three from *A. pisum* (*Buchnera*-ApTokyo, *Buchnera*-Ap5A, *Buchnera*-ApTuc7), and one each from *Schizaphis graminum*, *Baizongia pistaciae*, and *Cinara cedri* (*Buchnera*-Sg, *Buchnera*-Bp, *Buchnera*-Cc). Two were newly obtained, those for *Buchnera* of *Uroleucon ambrosiae*, and *Acyrthosiphon kondoi* (*Buchnera*-Ua and *Buchnera*-Ak).

### Two newly sequenced *Buchnera* genomes

The genomes of *Buchnera*-Ak and *Buchnera*-Ua were completed at high depth of coverage (>100X, Table S1), and their general features were compared with previously sequenced *Buchnera* genomes (Table 1). Both genomes contained a single chromosome and two plasmids, pLeu and pTrp, as observed in other *Buchnera* of aphid species within the subfamily Aphidinae. The relative depths of coverage of chromosomal sequences and plasmids by *Illumina* reads suggest that both plasmids are present at 2 to 3 copies per chromosome in *Buchnera*-Ak and at about 0.3 to 0.4 copies per chromosome in *Buchnera*-Ua. Since *Buchnera* cells are highly polyploid [18], plasmids can be present in lower copy number than the main chromosome and still present in every cell.

**Table 1:** Features of completed *Buchnera aphidicola* genomes.

	*U. ambrosiae*	*A. kondoi*		*A. pisum*		*S. graminum*	*B. pistaciae*	*C. cedri*
	Ua	Ak	Tokyo	5A	Tuc7	Sg	Bp	Cc
Chromosome	615,380	641,794	640,681	642,122	641,895	641,454	615,980	416,380
G+C %	24.1	25.7	26.3	26.3	26.3	25.3	25.3	20.1
Plasmids	2	2	2	2	2	2	0	1
CDS	529	559	556	558	559	548	504	353
rRNA operons^1^	1	1	1	1	1	1	1	1
tRNAs	32	32	32	32	32	32	32	31
ncRNAs^2^	3	3	3	3	3	3	3	3
Homopolymeric frameshifts	8	10	11	13	12	18	1	3
Total pseudogenes	5	8	9	5	5	17	8	4
1 mutation	1	1	4	0	0	2	1	1
2 mutations	1	0	0	0	0	4	0	1
≥3 mutations	3	7	5	5	5	11	7	2

1 *rrs* is separated from *rrl-rrf*.

2 Already annotated: does not include newly discovered RNAs in the present analysis.

The deep sequence coverage revealed several polymorphic sites within both *Buchnera*-Ak and *Buchnera*-Ua, including both single nucleotides changes and single base indels and totaling 13 and 15 sites in the two species (Table S2). Polymorphisms were unexpected since both samples were derived from lab cultures descended from a single founding female and bottlenecked every few generations during rearing. Also, intrastrain polymorphism was not previously detected in deep sequencing of *Buchnera*-Ap strains [17], although it was recently detected in *Buchnera*-Sg [19].

### Phylogenetic reconstruction

**Figure 1:**
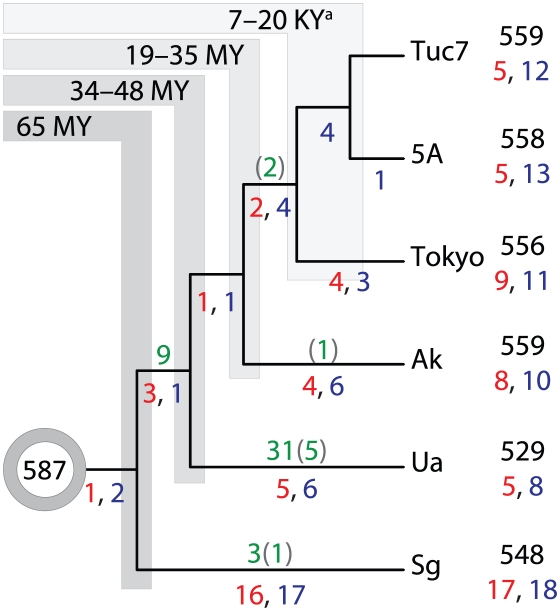
Gene erosion and loss along lineages of *Buchnera*. Column at right contains numbers of retained genes (black), numbers of pseudogenes caused by base substitutions or multiple mutations (red) and those with single homopolymeric frameshifts (blue). An ancestral gene set, as inferred from contemporary genomes, consisted of 587 intact coding genes. Traced along each lineage are the two classes of pseudogenes (red and blue), and gene losses (green) with deletions of inferred pseudogenes shown in parentheses. ^a^Age estimate for divergence of three *A. pisum* lineages based on [17].

To achieve our aim of analyzing IGS to detect gene decay and conserved functional elements, we needed a phylogeny for our strains. We reconstructed phylogenies based on sequences of proteins inferred from the sequenced genomes; these were consistent both with those based on 16S rRNA sequences for a larger set of species (Figure S1) and with current knowledge of aphid phylogenetic relationships [20]–[21]. Relationships were unambiguous except that the branching order of the 3 *Buchnera*-Ap genomes could not be resolved unequivocally due to the recency of their divergence. We also estimated divergence dates within the Aphidinae using the consensus phylogeny (Figure 1).

### Inactivation and loss of coding genes during *Buchnera* evolution

Using the two new genome sequences for *Buchnera*-Ak and *Buchnera*-Ua, we were able to detect and reconstruct gene content changes on lineages within the clade containing *Buchnera*-Ap strains, *Buchnera*-Ak, *Buchnera*-Ua, and *Buchnera*-Sg, corresponding to *Buchnera* of the subfamily Aphidinae (Table S3). These changes were scored as either losses (no gene remnants detectable) or inactivations (recognizable pseudogenes remain) (Figure 1).

Compared to the *Buchnera* of other Aphidinae species, *Buchnera*-Ua has lost more genes due to both deletions (up to 5 kb) and to the inactivation of genes that are intact in other *Buchnera* genomes. The major deletions include genes underlying the membrane-associated complex involved in oxidative stress (*rnfABCDGE*), DNA repair (*mutT, mutS, mutL*), pyrimidine biosynthesis (*pyrC, pyrD, pyrBI*), pantothenate biosynthesis (*panBC*), spermidine biosynthesis (*speED*), ornithine biosynthesis (*argECB, argA, argD*) and a transcriptional dual regulator (*hns*).

Differences in gene content between *Buchnera*-Ak and *Buchnera*-Ap are fewer (Table S3). Notably the major regulator of *metA* in methionine biosynthesis, *metR*, is intact in *Buchnera*-Ak and a pseudogene in *Buchnera*-Ap, suggesting that *Buchnera*-Ak may retain substrate-based activation of *metE* transcription, as observed previously in *Buchnera*-Sg which also retains intact *metR* [14]. An uninterrupted copy of Asparaginase I (*ansA*) was also identified in *Buchnera*-Ak, which is responsible for the interconversion of aspartate and ammonia into asparagine. In light of the current picture of metabolic cooperation between *Buchnera* and *A. pisum* [22], this gene could influence amino acid metabolism and nitrogen recycling in *A. konodi*. Also, *Buchnera*-Ak has undergone inactivation of the regulator *hns*, for which the ORF is intact in *Buchnera*-Ap.

The majority (42/69) of pseudogenes identified in these four genomes are restricted to single genomes and are caused by mutations in homopolymeric runs, as observed previously for pseudogene formation in strains of *Buchnera*-Ap [17]. In several instances, the identical gene has been inactivated independently in different lineages and through different mutations (Table S3). As one example, the gene *murF*, which is involved in biogenesis of the cell envelope, is intact only in *Buchnera*-ApTokyo but is inactivated by homopolymeric frameshifts in *Buchnera*-Ap strains 5A and Tuc7, *Buchnera*-Ak, *Buchnera*-Ua, *Buchnera*-Sg. This situation implies that there were four independent inactivations of this gene in the different lineages. The genes neighboring *murF*, *murE* and *mraY* are also inactivated by homopolymeric frameshifts in *Buchnera*-Sg. A second such example involves the global regulator *hns* mentioned above, which is intact in the three closely related *Buchnera*-Ap genomes but is deleted or pseudogenized in the other sequenced *Buchnera* genomes, implying three independent losses. Our comparisons reveal fourteen other cases in which orthologs, spanning a variety of functional categories, are independently inactivated in different *Buchnera* genomes, indicating that these genes are prone to repeated inactivation (Table S3).

### Patterns of conservation in intergenic spacers

**Figure 2:**
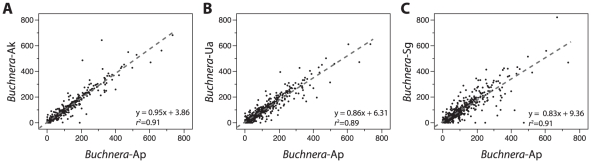
Conservation of IGS lengths among *Buchnera* from Aphidinae hosts.

Orthologous IGSs were compared for *Buchnera* of the four species of Aphidinae, and a strong association was detected between the IGS lengths of *Buchnera*-Ap and those of each of the others (Figure 2). The relationship among spacer lengths is even stronger for more closely related species pairs, such as *Buchnera*-Ap and *Buchnera*-Ak.

A phylogenetic footprinting approach [23]–[24] was utilized to identify sequence blocks within IGSs that might be conserved as functional elements, such as riboswitches, binding sites affecting transcription, transcriptional terminators, and sRNAs. *Buchnera*-Sg, *Buchnera*-Ua, *Buchnera*-Ak, and *Buchnera*-Ap contain from 580 to 621 genes, and total of 537 orthologous IGS regions were identified for these species. Of these, we focused our analysis on the 336 IGSs with alignments of at least 50 nucleotides and with no zero or negative length spacers (overlapping genes). The IGSs in these genomes have an average base composition of only 14–16% G+C, whereas the mean G+C contents of coding regions range from 25.3–27.3%.

These spacers can be divided according to whether they are flanked by genes transcribed in tandem (225 IGSs), convergently (42 IGSs), and divergently (70 IGSs), respectively (Figure S2). The average sequence identity for the three categories is similar, ranging from 51.3–53.9%, but the average alignment length is substantially greater for IGSs of divergently oriented genes, at 288 nucleotides, compared to 160 and 136 nucleotides for the tandem and convergent categories. The correlation of spacer lengths is the greatest for the divergent category and lower for the other two categories; for the species pair *Buchnera*-Sg–*Buchnera*-Ap, the pairwise correlation of IGS lengths are 0.78 for tandem, 0.80 for convergent, and 0.87 for divergent IGSs (Pearson's *r*).

**Figure 3:**
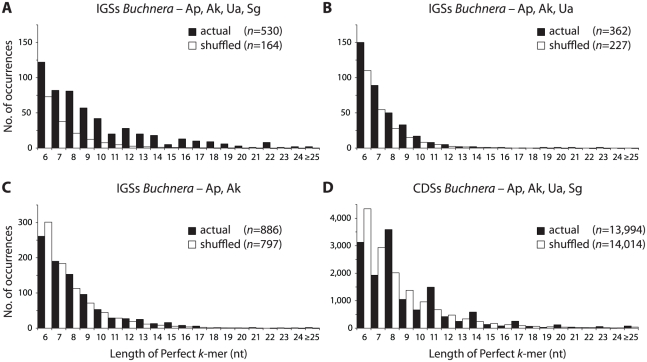
Actual and expected distributions of conserved *k*-mers in IGSs and CDs. Distribution of perfectly matching stretches of nucleotides (*k*-mers) of at least 6 nucleotides in alignments of (A) IGSs of *Buchnera* from *S. graminum*, *U. ambrosiae*, *A. kondoi*, and *A. pisum*, (B) IGSs of *Buchnera* from *U. ambrosiae*, *A. kondoi*, and *A. pisum*, showing only the additional *k*-mers conserved only in these three genomes, (C) IGSs of *Buchnera* from *A. kondoi* and *A. pisum*, showing only the additional *k*-mers conserved only in these two genomes, (D) CDSs for *S. graminum*, *U. ambrosiae*, *A. kondoi*, and *A. pisum*.

To detect whether the IGSs contain conserved regions of possible functional significance, we examined the occurrence of perfectly conserved *k*-mers of at least five nucleotides in the 4-taxon alignments (hereafter referred to as “conserved *k*-mers”). The length distribution of conserved *k*-mers was compared to that of the randomly shuffled alignments. The two distributions depart, with an increased incidence of conserved *k*-mers for the observed IGS alignments than would be expected by chance (Figure 3).

The elevation of conserved *k*-mers relative to the random expectation was far more pronounced for IGSs than for protein-coding regions. This observation can be understood as the result of the saturation of synonymous sites across the evolutionary divergence represented by these four *Buchnera* species. Even though many proteins have high conservation of amino acid sequences, synonymous site differences reduce the number of conserved *k*-mers to only slightly higher than the number expected by chance. The pattern observed in coding regions, an excess of (3n−1) *k*-mers, is a result of this saturation at synonymous (third) positions (Table 2). Despite the large synonymous divergences among *Buchnera* strains, we find that *k*-mers≥6 nucleotides make up 26% of the alignment corresponding to the 473 coding sequences (126,973 nt/487,127 nt); however, such *k*-mers constitute only 8.4% of the alignment corresponding to the IGSs (5,068 nt/60,673 nt). This difference reflects the fact that coding regions are generally more conserved, due to strong conservation at nonsynonymous sites, than IGS regions. However, when conservation does occur in IGS regions, it often involves runs of 6 or more nucleotides, which is not usually true in coding regions.

**Table 2:** Synonymous and nonsynonymous divergences averaged across orthologous protein-coding genes of *Buchnera*-Ap, *Buchnera*-Ak, *Buchnera*-Ua, and *Buchnera*-Sg.

*Ka\Ks* *	Ap			Ak			Ua			Sg		
**Ap**		–		1.47	±	1.94	3.96	±	10.10	7.93	±	19.59
**Ak**	0.0722	±	0.044		–		3.77	±	11.64	6.01	±	14.32
**Ua**	0.1200	±	0.068	0.1153	±	0.065		–		11.06	±	24.02
**Sg**	0.1491	±	0.087	0.1433	±	0.081	0.1657	±	0.093		–	

*Average (± std. dev.) of ML estimates of *Ka* (lower) and *Ks* (upper) for 473 CDS.

The excess of conserved *k*-mers was also more pronounced for *k*-mers conserved across the 4-taxon alignment than for *k*-mers conserved exclusively within the shallower alignments of 3 or 2 taxa (Figure 3). The opposite would be expected if the excess of conserved *k*-mers in IGS regions merely reflected recent ancestry reflected in high sequence similarity even in neutral regions. Instead, the robust conservation even at deeper divergences, which are fully saturated for changes at neutral sites, favors a major role of purifying selection in maintaining these sequences.

For IGSs, the excess of conserved *k*-mers was observed across all length categories, from 5 to 22 nucleotides. It is possible that the excess of conserved *k*-mers is merely the by-product of low-complexity, A−T-rich intergenic spacers, however two lines of evidence oppose this view. (1) Of the 336 IGS regions, 232 contained at least one conserved *k*-mer of at least 5 nucleotides, whereas in shuffled alignments with the same base compositions, only 137 IGSs contained a conserved *k*-mer of at least 5 nucleotides. (2) Although many (320/775) *k*-mers are entirely composed of As and Ts, longer *k*-mers tend to be more GC rich, and the overall distribution of GC compositions of *k*-mers departs from that of IGSs as a whole (Figure S3). This suggests that most IGSs of length greater than 50 nucleotides contain functional regions that are highly constrained.

Considering the three IGS categories based on orientation of flanking genes, the excess of conserved *k*-mers was greater for IGSs between divergently transcribed genes, at 1.44 *k*-mers per 100 nucleotides compared to IGSs between tandem or convergently transcribed genes (1.07 and 1.26 IGS per 100 nucleotides). Because the IGSs between divergently transcribed genes were also longer on average, they had substantially more conserved *k*-mers per IGS.

### Recognizing functional elements within intergenic spacers

There is additional evidence of some type of functional element within the majority of the 232 IGSs with conserved *k*-mers. First, 78 IGSs contained one or more perfectly conserved, or nearly perfectly conserved, Shine-Dalgarno (SD) sequence (consensus AGGAG) for the four taxon alignment (*n* = 88), indicating that such alignments can be used to improve annotation of SD sequences. In addition, we detected 55 putative transcriptional terminators. Of these, most occur in orthologous locations of validated (*n* = 11) or predicted (*n* = 25) transcriptional terminators in *E. coli* K12 (**EcoCyc** and **TransTermHP**). Four of the conserved IGSs corresponded to previously identified RpoH (heat shock sigma factor, or Sigma-32) binding sites upstream of *dnaKJ*, *grpE*, *groESL*, and *ibpA* [13] with near-perfect conservation among the four taxa. In addition, our analyses detected a putative RpoH binding site upstream of *rpoD*. Four regions with conserved *k*-mers contained sRNAs previously annotated in *Buchnera* and correspond to the 4.5S RNA component of the signal recognition particle (*ffs*), the catalytic subunit of RNAse P (*rnpB*), transfer-messenger RNA (*ssrA*), and 5S ribosomal RNA (*rrf*). Using **RNAz** another 12 putative sRNAs were detected, including two with positional orthology to *tpke11* and *sraA* of *E. coli* K12.

**Figure 4:**
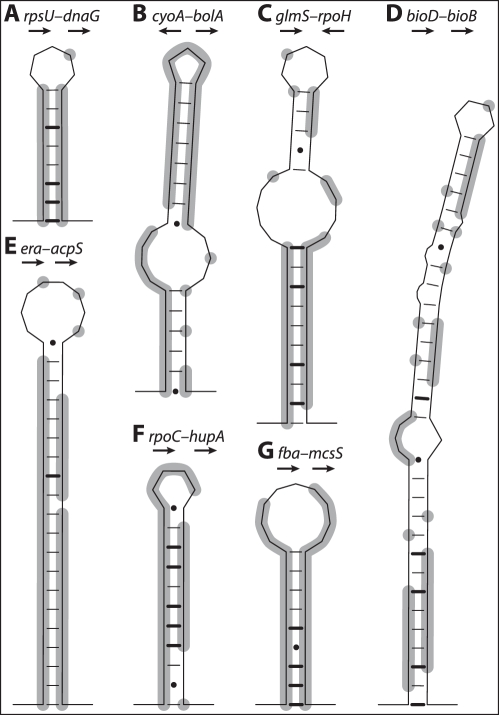
Structural predictions for IGSs with conserved elements across *Buchnera*-Ap, Ak, Ua, and Sg. Genes flanking each IGS and their relative orientation are shown. Positions identical in all four *Buchnera* genomes are highlighted in grey. Bonds between cytosine-guanine (C–G) pairs are shown as bold lines, adenine-thymine (A–T) pairs as thin lines, and guanine-thymine (G–T) pairs shown as dots (•).

Thus, of the 232 IGSs with at least one conserved *k*-mer, a total of 129 encode regions for which a function can be inferred based either on their homology sequences in *E. coli* or on their structural features. Because **RNAz** cannot be used to analyze sequences of >75% A+T content and SD sequences are quite short (5 nt), 93 IGS alignments with *k*-mer blocks (see Materials and Methods) were reanalyzed for additional functional elements. The IGS alignments were trimmed down to the boundaries of the conserved regions and analyzed with **RNAalifold** to detect secondary structural properties. Of the 93 IGSs examined, 61 had significant secondary structures (*t*-test, *p*<0.05), and many formed conserved hairpin structures although some contained internal bulges (Figure 4). Some of these may represent transcriptional terminators; however, it is possible, particularly among the longer conserved regions with more complex structural predictions, that these regions are expressed as sRNAs. Moreover, 22 of these 93 conserved IGSs are found between divergently arranged CDS, raising the possibility that the conserved regions act as binding sites or possibly leader sequences influencing transcription or translation of the mRNAs.

Further analyses of all the IGSs, including several conserved in only three taxa due to deletions in individual *Buchnera* lineages, revealed another 12 conserved transcriptional terminators and 10 well-conserved Shine-Dalgarno sequences. Of the total of 67 transcriptional terminators, 29 were also retained in *Buchnera*-Bp and/or *Buchnera*-Cc. The majority of the transcriptional terminators shared by all six genomes, however, do not show absolute conservation of the primary sequence. When the IGSs of *Buchnera*-Bp and *Buchnera*-Cc are considered with those from the four other genomes, only 281 of the 537 orthologous spacers are conserved, and only 25 *k*-mers≥6 nt are identified. This includes *k*-mers that coincide with IGSs containing putative RNA secondary structures (*n* = 12), Sigma-32 binding sites (*n* = 3) and a single transcriptional terminator (*n* = 1).

## Discussion

The genomes of obligate bacterial symbionts are highly reduced in size and gene repertoires due to a combination of factors. First, the symbiotic lifestyle in a nutrient-rich host environment renders numerous genes superfluous, allowing the inactivation of many previously functional regions [10]. In addition, the dynamics of symbiont transmission to new hosts involve severe restrictions in population size and impose clonality, thereby reducing the efficacy of selection and fostering the accumulation of deleterious mutations [25]–[27]. When combined with the pervasive mutational bias in bacteria in which deletions outnumber insertions, regions that are not under strong selective constraints erode and are eventually lost, leading to small and compact genomes [6]–[7].

Due to difficulties in defining the variety or size of functional elements that might potentially occur within intergenic spacers, the most common comparative methods, such as *Ka*/*Ks* ratio tests, are not useful for differentiating spacers (or those portions of spacers) that are functional from those that are inert. This problem is further compounded by the extreme AT-richness of symbiont genomes, which can cause erroneous results from motif-finding and structural algorithms. Therefore, we tested the degree of conservation for a series of short sequences (*k*-mers) across orthologous regions from *Buchnera aphidicola* of varying degrees of phylogenetic relatedness (Figure S1). To enhance the strength and validity of these tests, we generated complete *Buchnera* genome sequences for two aphid species (*Buchnera*-Ua and *Buchnera*-Ak), which provided information at intermediate levels of relatedness.

Based on the presence of identical *k*-mers within orthologous regions across genomes, intergenic spacers (IGSs) contain an excess of conserved *k*-mers relative to protein-coding regions, indicating most IGSs contain some type of functional elements. Because these analyses require that *k*-mers be identical, many of the functional regions within IGSs are considerably longer than the associated *k*-mers but do not show perfect conservation along their entire lengths. Also, we found that conserved *k*-mers are often located near one another in the same IGS (*k*-mer blocks), suggesting that they are parts of the same functional element.

Orthologous IGSs exhibit not only sequence conservation, as reflected in the elevated numbers of identical *k*-mers, but also substantial conservation of length across *Buchnera* genomes (Figure 2). The similarity in spacer length among *Buchnera* lineages could be attributable either to selection on the functional elements within spacers or simply to shared ancestry, such that genomes retain ancestral spacer lengths due to lack of time for mutations affecting length to occur. However, the latter explanation is excluded by the observation that DNA from inactivated functional elements is largely eliminated across the time scales corresponding to divergence of these lineages (up to 70 MYA). For example, along the lineage leading to *Buchnera*-Ua, DNA for 36 genes or pseudogenes was eliminated, with only 13 pseudogenes recognizable in the genome. Thus, the conservation of spacer lengths is largely attributable to functional constraints.

**Figure 5:**
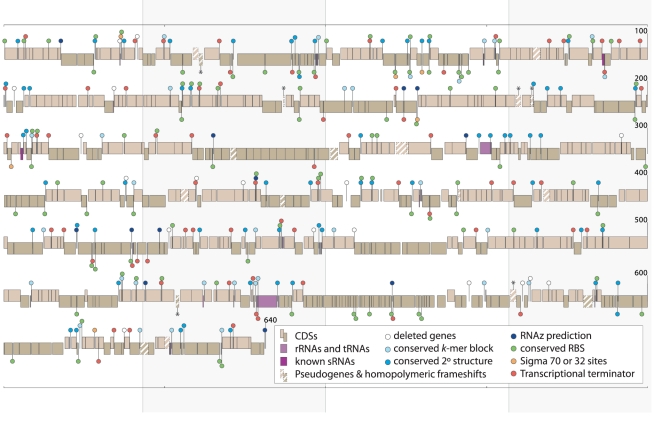
IGS features in the *Buchnera*-ApTokyo genome. Staggered boxes represent annotated genes located on the forward (higher) or reverse (lower) strands. The majority of the 336 long intergenic regions contain conserved sequence blocks, many with inferred functions, as indicated by the color-coded ball-and-sticks. Asterisks (*) denote newly identified pseudogenes in *Buchnera*-ApTokyo. Size scale is in kb.

**Figure 6:**
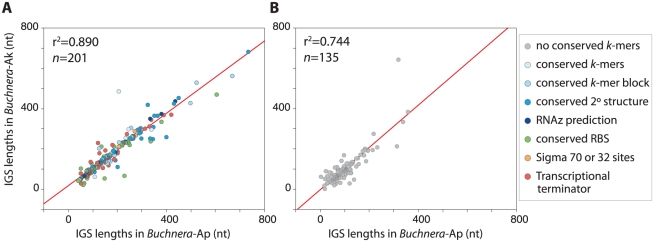
Lengths of orthologous IGSs in *Buchnera*-Ap and *Buchnera*-Ak. Intergenic spacers (IGSs) are divided into those (A) with and (B) without conserved elements and color-coded according to their inferred functions and/or presence of conserved *k*-mers. Conserved “*k*-mer blocks” are defined as ≥2 *k*-mers less than 30 nt apart or *k*-mers≥10 nt.

Taken as a whole, our analyses established that at least 201 of the 336 IGSs of at least 50 nucleotides in length encode functional elements (Figure 5). Some of those remaining might harbor functional sequences that do not rely on conserved motifs; for example, the standard sigma-70 binding sites (RpoD sites) have a relatively weak and AT-rich consensus sequence in *E. coli* (TTGaCann [15]–[19] nnTAtAaT). However, many IGSs probably consist of decaying pseudogenes. We note that the IGSs that do not contain conserved *k*-mers have more often undergone changes in length during the divergence of *Buchnera*-Ap and *Buchnera*-Ak (Figure 6), as expected for functionally inactive regions (Fisher's *r*-to-*z* transformation, *p*<0.0001).

Our analyses focused on a cluster of *Buchnera* corresponding to aphids in the Aphidinae and including the focal species, *Buchnera*-Ap. This depth of comparison provided sufficient divergence to detect conserved elements not due to recent shared ancestry. Searching for conserved IGS regions in the more distant genomes of *Buchnera*-Cc and *Buchnera*-Bp did reveal some of the same elements. However, these were relatively few due to the reduction in number of clearly identifiable orthologous IGSs (reflecting divergence in gene repertoires) and to the lack of strict conservation of sequence for stretches corresponding to k-mer lengths. The intermediate level of comparison enabled by the newly sequenced genomes was critical to detecting conserved elements.

In addition to the variation observed in intergenic spacers, the process of genome reduction is also expected to cause differences in the gene catalogs and the pseudogene contents of these genomes. Among *Buchnera* from aphids in the subfamily Aphidinae, the newly sequenced *Buchnera*-Ua encodes the fewest protein-coding genes. Certain of these gene losses in *Buchnera*-Ua may reflect changes in its nutritional ecology, related to the host plant, or a greater reliance upon the host or presence of an additional symbiont. The composition of phloem sap ingested by *U. ambrosiae* feeding on one of its host plant (*Tithonia fruticosa*) contains very high amounts of arginine (25% of free amino acids) [28]; elevated arginine in the diet potentially has led to relaxation of selection for the maintenance of ornithine biosynthesis, resulting in the loss of that pathway in *Buchnera*-Ua. Alternatively these gene losses (*argECB, argA, argD*) may be influenced by increased metabolic cooperation between *U. ambrosiae* and *Buchnera*-Ua in that the activity of an aphid-derived ornithine aminotransferase (EC 2.6.1.13), involved in analogous functionality, was recently demonstrated to be up-regulated in the bacteriocytes of *A. pisum* [22].

The *Buchnera*-Ua genome also has lost the genes for pantothenate biosynthesis (*panBC*), possibly due to the transfer from dependence on *Buchnera* for pantothenate provisioning to dependence on another bacterial symbiont, *Hamiltonella defensa*, which is universally present in *U. ambrosiae* and closely related *Uroleucon* from North America. This *Uroleucon*-associated strain of *H. defensa* contains *panBCD* and appears to be a stable coevolving symbiont of this clade of *Uroleucon* species, along with *Buchnera* ([29], P. Degnan unpublished).

Ongoing gene erosion in *Buchnera* has resulted in 15 convergent cases of gene inactivation and loss along independent lineages of the Aphidinae. Although several of these events involve highly degraded or deleted genes (e.g., *ansA, hflD, hns*), nine involve inactivating mutations generated by an indel in a homopolymeric tract. Such mutations are common in endosymbiont genomes due to their highly biased base compositions and are commonly interpreted as ‘recent’ gene inactivations [17], [30]. In fact, between 10 and 70% of disrupted genes identified in *Buchnera* genomes result from indels occurring within homopolymeric repeats (Table 1). However, it has been demonstrated that mRNAs for an inactivated locus in *Buchnera* of the aphid *Rhopalosiphum padi* can be corrected by transcriptional slippage to yield functional proteins [31]. This phenomenon has been suggested to potentially play a role in regulating gene expression [31]. Therefore, while some convergent gene loss may be the result of independent inactivation events, reflecting low functional constraint, it is plausible that some of these mutations provide an alternative means of gene regulation in *Buchnera*.

Many of the spacers that do not contain functional elements are pseudogenes in various stages of decay, including some newly identifiable on the basis of comparisons between *Buchnera*-Ap, *Buchnera*-Ak and *Buchnera*-Ua (Figure 5). Although the symbiosis between *Buchnera* and aphids has existed for more than 150 million years and the ancestral *Buchnera* already had a highly reduced genome [11], the loss of genes has been ongoing during this period, even among strains confined to a single aphid host species, as observed for the *Buchnera*-Ap strains (Fig. 2 in [17]). The continuous production of new pseudogenes, and the resulting new intergenic spacers, is perhaps surprising given the long co-evolution and functional interdependence of the symbiont and host. However, *Buchnera* genomes are not nearly the smallest genomes found in symbiotic bacteria [32]–[33], implying that symbiotic bacteria are able to endure and compensate for continued gene loss.

## Materials and Methods

### Isolation and sequencing of *Buchnera* DNA

Two aphid species, *Acyrthosiphon kondoi* (blue alfalfa aphid) and *Uroleucon ambrosiae* (brown ambrosia aphid), were selected based on availability and on evolutionary relationships to aphid species for which *Buchnera* genome sequences were already available. Isofemale lines of *A. kondoi* and *U. ambrosiae* str. UA002 were established from single parthenogenic females collected in Tucson, AZ. The *A. kondoi* was collected from *Medicago sativa* (alfalfa) on 21 March 2007 and then reared on *M. sativa* in a growth chamber at 20°C under a 16 h light/8 h dark regime. The *U. ambrosiae* was collected from *Encelia farinosa* (brittlebush) on 18 March 2006 and maintained on *Tithonia rotundifolia* (Mexican sunflower) under ambient greenhouse conditions at ∼25°C with natural lighting.

Preparation of purified DNA from *Buchnera* of *A. kondoi* (*Buchnera*-Ak) and *Buchnera* of *U. ambrosiae* (*Buchnera*-Ua) was performed as in Charles and Ishikawa [34], with the following modifications. *Buchnera* cells from *A. kondoi* were isolated by initially homogenizing whole insects in Buffer A-250 (250 mM EDTA pH 8.0, 35 mM Tris pH 8.0, 25 mM KCl, 10 mM MgCl_2_, 250 mM sucrose; as in [35]) and filtering through a 100 µm nylon filter. The cells were centrifuged at 1,500× *g* and 4°C for 10 minutes, resuspended in fresh Buffer A-250 and then serially filtered through 20, 11, and 8 µm filters. Intact *Buchnera* cells were collected by a final centrifugation for 25 minutes at 1,500× *g* and 4°C. The DNA sample prepared for 454-pyrosequencing was immediately extracted using the PureGene Tissue Core Kit B (Qiagen). The sample prepared for *Illumina* sequencing was further purified using a Percoll density gradient prior to DNA extraction. Genomic DNA from *Buchnera*-Ua was isolated in a similar fashion as above but with the following changes: Buffer A-100 was used (100 mM EDTA pH 8.0, 35 mM Tris pH 8.0, 25 mM KCl, 10 mM MgCl_2_, 250 mM Sucrose), only 100, 20 and 11 µm filters were used, intact cells were treated with DNAse prior to DNA extraction, and the Percoll density gradient was not used.

Genomic DNA was submitted for standard 454 pyrosequencing although different sequencing strategies were employed depending on the particular template. *Buchnera*-Ak was sequenced with a half run of GS-FLX, and *Buchnera*-Ua was sequenced with one and a half runs of GS-Titanium. To correct any potential artifacts introduced by 454 sequencing, samples were also sequenced on the *Illumina* platform: *Buchnera*-Ak using a high-AT, amplification-free library for 60 cycles, and *Buchnera*-Ua using a standard library for 36 cycles.

### Genome assembly and annotation

Pyrosequencing reads were assembled using **Newbler** (v1.1.03.24 for *Buchnera*-Ak, v2.0.00.19 for *Buchnera*-Ua) with default parameters and exported for **Consed** (v19) [36]. Contigs were binned by **Blast** [37] similarity to the *Buchnera*-ApTokyo genome [15], and contaminating scaffolds were removed (e.g., aphid). The contigs were then ordered and merged in **Consed** using Sanger sequencing reads produced by amplified PCR products spanning the gaps (as in [38]). *Illumina* reads were used to correct the consensus using the Perl script “*addSolexaReads.pl*”, which implements **cross_match** and is distributed with **Consed**. The entire genomes were manually screened, and any remaining assembly artifacts corrected.

Gene predictions were performed with **Prodigal** (v1.10) [39], **tRNA-scan** (v1.23) [40] and **Blast** (v2.2.24+). Functions of identified genes were inferred from *E. coli* K12 orthologs and metabolic descriptions in **EcoCyc** [41]. Pseudogenes were identified as sequences showing significant homology to known genes but that possessed one or more inactivating mutations that resulted in truncation to <80% of the length of known homologs. Inactivations resulting from frameshifts in homopolymeric tracts were counted separately. We note that previous studies and genome annotations used criteria for pseudogene designation that were more [15] or less [17] stringent than that applied here, often resulting in differences in pseudogene counts between studies. Sequence polymorphisms were recognized as sites that had multiple reads for each of two alternative bases or for an insertion/deletion in both the *Illumina* and 454 datasets.

These genomes have been deposited in GenBank (*Buchnera*-Ak: CP002645–CP002647 and *Buchnera*-Ua: CP002648–CP002650).

### Comparative and molecular evolutionary analyses

A total of eight *Buchnera* genomes, including the two newly determined genome sequences for *Buchnera*-Ak and *Buchnera*-Ua were included in analyses. Previously available sequences include *Buchnera* that are obligate symbionts of *Acyrthosiphon pisum* (strains Tokyo, 5A, Tucson), *Schizaphis graminum*, *Cinara cedri* and *Baizongia pistaciae*; these symbionts are designated *Buchnera*-ApTokyo, *Buchnera*-Ap5a, *Buchnera*-ApTuc7, *Buchnera*-Sg, *Buchnera*-Cc, and *Buchnera*-Bp (Figure S1). Given the high similarity among the *Buchnera* genomes from *A. pisum* [17], our analyses often used only one of the three genomes, which was subsequently designated *Buchnera*-Ap. Complete *Buchnera* genomes were aligned with **MAUVE** [42], identifying shared orthologs. Conserved protein-coding genes were individually aligned with **MAFFT** [43] based on their amino acid sequences and then reverse-translated. For sets of orthologs containing one or more pseudogenes, intact genes were aligned as above, and pseudogenes were added secondarily and aligned manually. Estimates of pairwise divergence were calculated with **PAML** for each gene (codeml runmode = −2) [44]. Additionally, a phylogeny based on concatenated amino acid alignments was estimated in **RAxML** [45] utilizing 20 random sets of 50 proteins common to all eight *Buchnera* genomes (325 CDS), with dates estimated with **r8s** [46]. Best topologies and 100 bootstrap replicates for each data set were calculated using the CPREV amino acid substitution matrix and the gamma model of rate heterogeneity. Divergence dates were estimated for each of the 20 phylograms by cross-validating ages calculated from 10 replicates using the Penalized Likelihood (PL) method and Truncated Newton (TN) algorithm with a gamma rate distribution. The twenty tree topologies generated for each data set were virtually identical, except for the placement of the three *Buchnera*-Ap genomes, which differed in very few amino acid residues [17]. Maximum-likelihood estimates indicate a divergence between the genera *Uroleucon* and *Acyrthosiphon* occurring 41±7 MYA and the split between the two *Acyrthosiphon* species occurring 27±8 MYA (Figure 1). These ages represent averages from the 20 tree topologies using a mean fixed divergence time of 65 MYA between the Aphidini and Macrosiphini [47]. Estimates using a constrained range (50–70 MYA) or fixed minimum or maximum divergence dates were not markedly different.

For comparative purposes a 16S ribosomal RNA (rRNA) phylogeny of *Buchnera aphidicola* was generated using available sequences in GenBank. Briefly, full-length sequences were downloaded (>1,200 nt), aligned with **MAFFT** and analyzed with **MrBayes** (v3.1.2) [48]. The topology and posterior probabilities were co-estimated from two independent runs, each run with four chains progressing for 10 million generations and using a burn-in equal to 5% of the saved trees. Likelihood model parameters were set to six nucleotide rate categories, which varied according to the gamma distribution and included a proportion of invariant sites.

### Analysis of intergenic spacers for non-coding elements

Intergenic spacers (IGSs) were considered to be orthologous for different *Buchnera* genomes if flanked by orthologous genes in genomes being compared. Orthologous *Buchnera* IGSs were aligned with **MAFFT** and compared across species to identify sequence blocks containing conserved elements possibly indicating functional roles (transcriptional terminators, sRNAs, *etc.*). These analyses were conducted at two phylogenetic levels: across *Buchnera* of Aphidinae (*Buchnera*-Sg, *Buchnera*-Ua *Buchnera*-Ak, *Buchnera*-Ap) and across all sequenced *Buchnera* (including *Buchnera*-Cc and *Buchnera*-Bp). Although these comparisons only included the Tokyo strain of *Buchnera*-Ap, the patterns were virtually identical when the other *Buchnera*-Ap strains are included. We searched IGS alignments for perfectly conserved nucleotide stretches (*k*-mers) of greater than five nucleotides in length across the genomes of interest. The results were compared to random sequences that were obtained for each IGS alignment by shuffling with **Multiperm** (v0.9.4) [49].

Conserved IGSs were then analyzed by a variety of means to identify possible functional elements, which were then collated with the *k*-mer results. Computational detection of Shine-Dalgarno sequences (ribosome binding sites) was carried out using **RBSfinder** [50] and of *rho*-independent transcriptional terminators using **TransTerm-HP** (v2.07) [51] with default parameters. *Buchnera* IGSs were interrogated directly for confirmed *E. coli* K12 sRNAs (*n* = 83) using **Blastn** and positional homology. Subsequent identification of potentially novel sRNAs was performed in a two-step process. First, all IGSs alignments were analyzed with **RNAz** (v1.0) [52] for conserved thermodynamically stable RNAs. However, **RNAz** only works reliably for sequences of >25% G+C, and the mean G+C% of *Buchnera* IGS is ∼15%. Therefore, remaining highly conserved IGSs lacking identifiable functional domains and “*k*-mer blocks” (≥2 *k*-mers less than 30 nt apart or *k*-mers ≥10 nt) were analyzed with **RNAalifold** (v1.8.4) [53] for signatures of conserved secondary structure. Then individual alignments were randomly shuffled 100 times with the Perl script “*rnazRandomizeAln.pl*” distributed with **RNAz**, and the presence of possible secondary structures was determined with **RNAalifold**. A *t*-test was used to determine if the predicted free energy (kcal/mol) of the actual alignment was significantly less than the distribution of values calculated for the shuffled alignments. We note that the high A+T contents of *Buchnera* sequences were problematic for some motif finders, such as **AlignAce** (data not shown). All of the identified conserved elements are listed with their genome coordinates in the supporting information (Table S4).

## Supporting Information

Figure S1 Phylogenies of aphids based on *Buchnera* gene and protein sequences. (A) Broad aphid phylogeny generated from *Buchnera* 16S rRNA sequences and (B) narrow phylogeny based on sets of 50 protein sequences from sequenced *Buchnera* genomes. Phylogenies are consistent with one another and with views of aphid phylogenetic relationships. Posterior probabilities (16S rRNA phylogeny) and bootstrap values (protein phylogeny) <75% are shown in gray, and nodes with <50% support are collapsed.
(PDF)

Figure S2 Influence of flanking gene orientation on IGSs. Lengths and sequence conservation of orthologous IGSs in *Buchnera*-Ap and *Buchnera*-Sg where flanking genes are arranged (A) in tandem, (B) convergently or (C) divergently.
(PDF)

Figure S3 Evaluation of the base composition of conserved *k*-mers. (A) The distribution of base compositions for *k*-mers are compared to entire IGS from *Buchnera*-Ap and (B) compared by *k*-mer lengths. In (B) the size and color of spheres denote the relative occurrence of *k*-mers of a particular size and A+T content.
(PDF)

Table S1 Assembly statistics for newly sequenced *Buchnera aphidicola* genomes.
(XLS)

Table S2 Table of validated sequence polymorphisms identified in newly sequenced *Buchnera aphidicola* genomes.
(XLS)

Table S3 Summary list of genes inactivated or lost in *Buchnera* genomes of the Aphidinae.
(XLS)

Table S4 Descriptions and locations of conserved IGS elements.
(XLS)
