# Supplementary material for: Sequence Conservation and Functional Constraint on Intergenic Spacers in Reduced Genomes of the Obligate Symbiont Buchnera
Source: PLoS Genet. 2011 Sep 1;7(9):e1002252. doi: 10.1371/journal.pgen.1002252 (PMC3164680; doi:10.1371/journal.pgen.1002252)
Supplement: Figure S1 — Phylogenies of aphids based on Buchnera gene and protein sequences. (A) Broad aphid phylogeny generated from Buchnera 16S rRNA sequences and (B) narrow phylogeny based on sets of 50 protein sequences from sequenced Buchnera genomes. Phylogenies are consistent with one another and with views of aphid phylogenetic relationships. Posterior probabilities (16S rRNA phylogeny) and bootstrap values (protein phylogeny) <75% are shown in gray, and nodes with <50% support are collapsed. (PDF) [file pgen.1002252.s001.pdf]

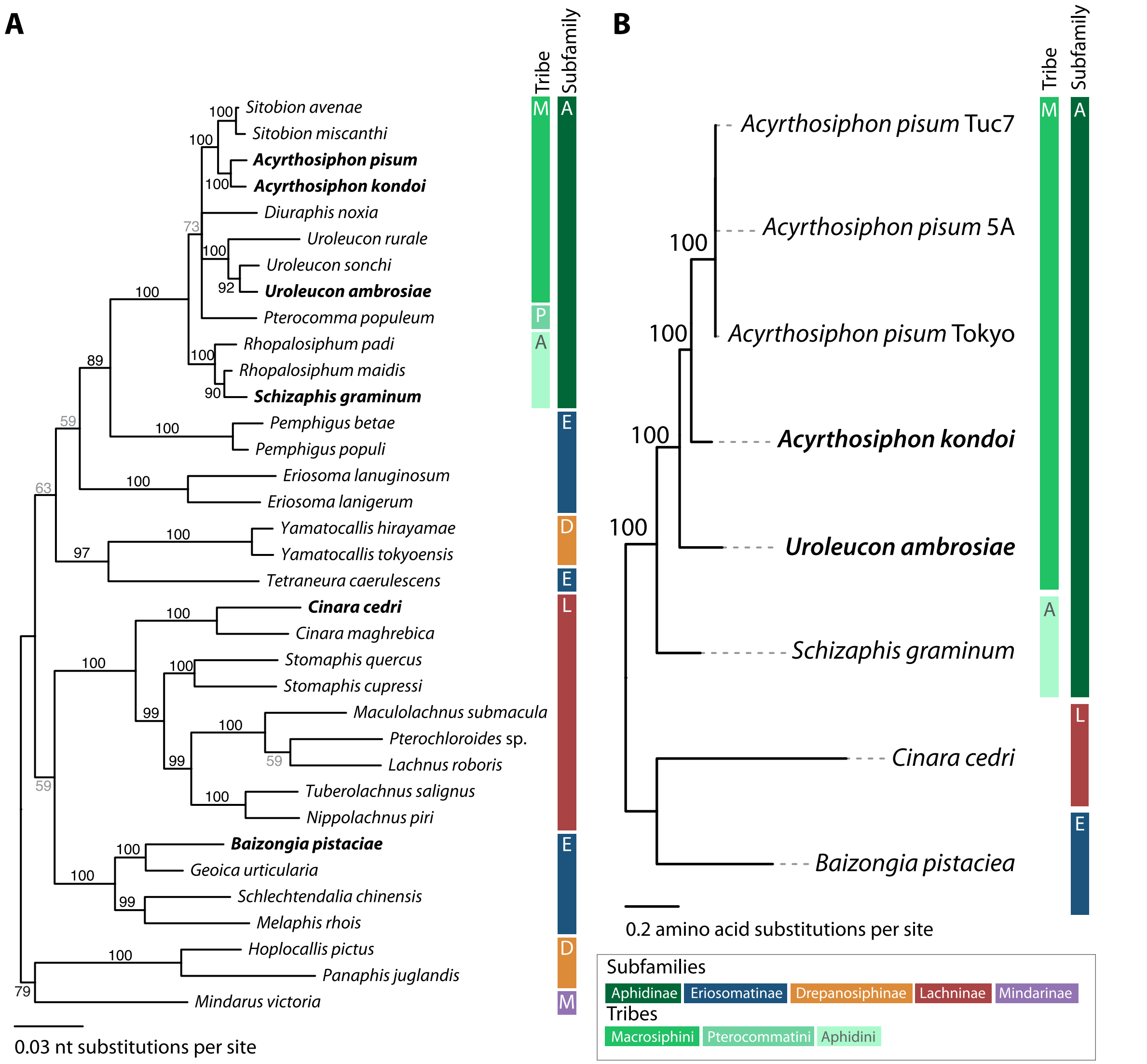
A
Tribe
Subfamily
Sitobion avenae
Sitobion miscanthi
Acyrthosiphon pisum
Acyrthosiphon kondoi
Diuraphis noxia
Uroleucon rurale
Uroleucon sonchi
Uroleucon ambrosiae
Pterocomma populeum
Rhopalosiphum padi
Rhopalosiphum maidis
Schizaphis graminum
Pemphigus betae
Pemphigus populi
Eriosoma lanuginosum
Eriosoma lanigerum
Yamatocallis hirayamae
Yamatocallis tokyoensis
Tetraneura caerulescens
Cinara cedri
Cinara maghrebica
Stomaphis quercus
Stomaphis cupressi
Maculolachnus submacula
Pterochloroides sp.
Lachnus roboris
Tuberolachnus salignus
Nippolachnus piri
Baizongia pistaciae
Geoica urticularia
Schlechtendalia chinensis
Melaphis rhois
Hoplocallis pictus
Panaphis juglandis
Mindarus victoria
0.03 nt substitutions per site
B
Acyrthosiphon pisum Tuc7
Acyrthosiphon pisum 5A
Acyrthosiphon pisum Tokyo
Acyrthosiphon kondoi
Uroleucon ambrosiae
Schizaphis graminum
Cinara cedri
Baizongia pistaciea
0.2 amino acid substitutions per site
Subfamilies
Aphidinae
Eriosomatinae
Drepanosiphinae
Lachninae
Mindarinae
Tribes
Macrosiphini
Pterocommatini
Aphidini
