# Supplementary figures and images for: Sequence Conservation and Functional Constraint on Intergenic Spacers in Reduced Genomes of the Obligate Symbiont Buchnera
Source: PLoS Genet. 2011 Sep 1;7(9):e1002252. doi: 10.1371/journal.pgen.1002252 (PMC3164680; doi:10.1371/journal.pgen.1002252)

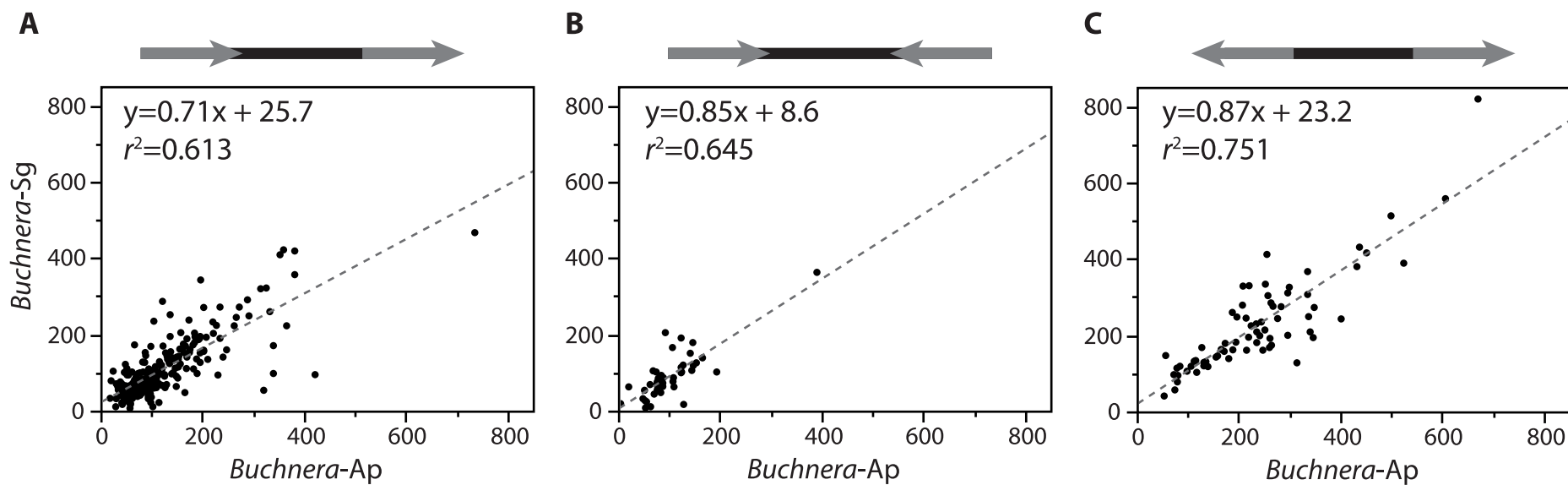

|                               |      |      |      |
|-------------------------------|------|------|------|
| No. of IGSs                   | 225  | 41   | 70   |
| Average % identity            | 51.3 | 52.8 | 53.9 |
| Average no. of <i>k</i> -mers | 1.7  | 1.7  | 4.2  |
| Average align. length         | 160  | 136  | 288  |
| <i>k</i> -mer per 100 nt      | 1.07 | 1.26 | 1.44 |

Supplement: Figure S2 — Influence of flanking gene orientation on IGSs. Lengths and sequence conservation of orthologous IGSs in Buchnera-Ap and Buchnera-Sg where flanking genes are arranged (A) in tandem, (B) convergently or (C) divergently. (PDF) [file pgen.1002252.s002.pdf]

**A**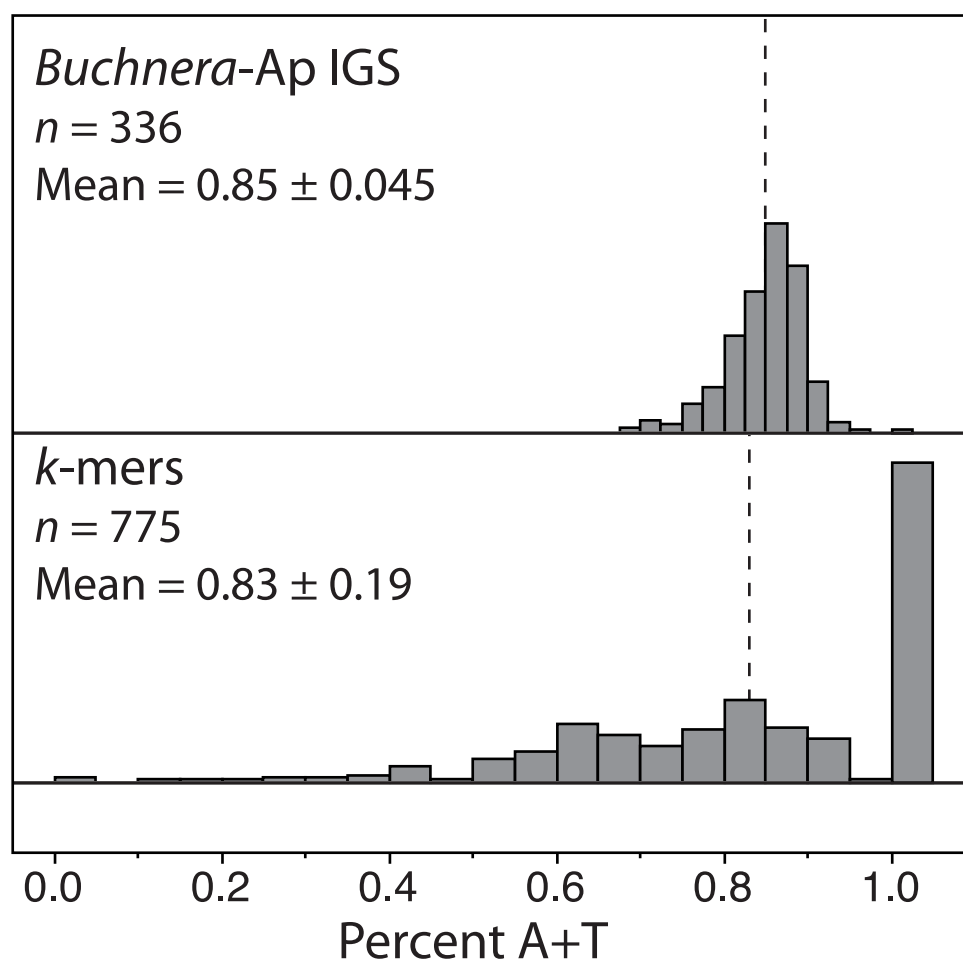**B**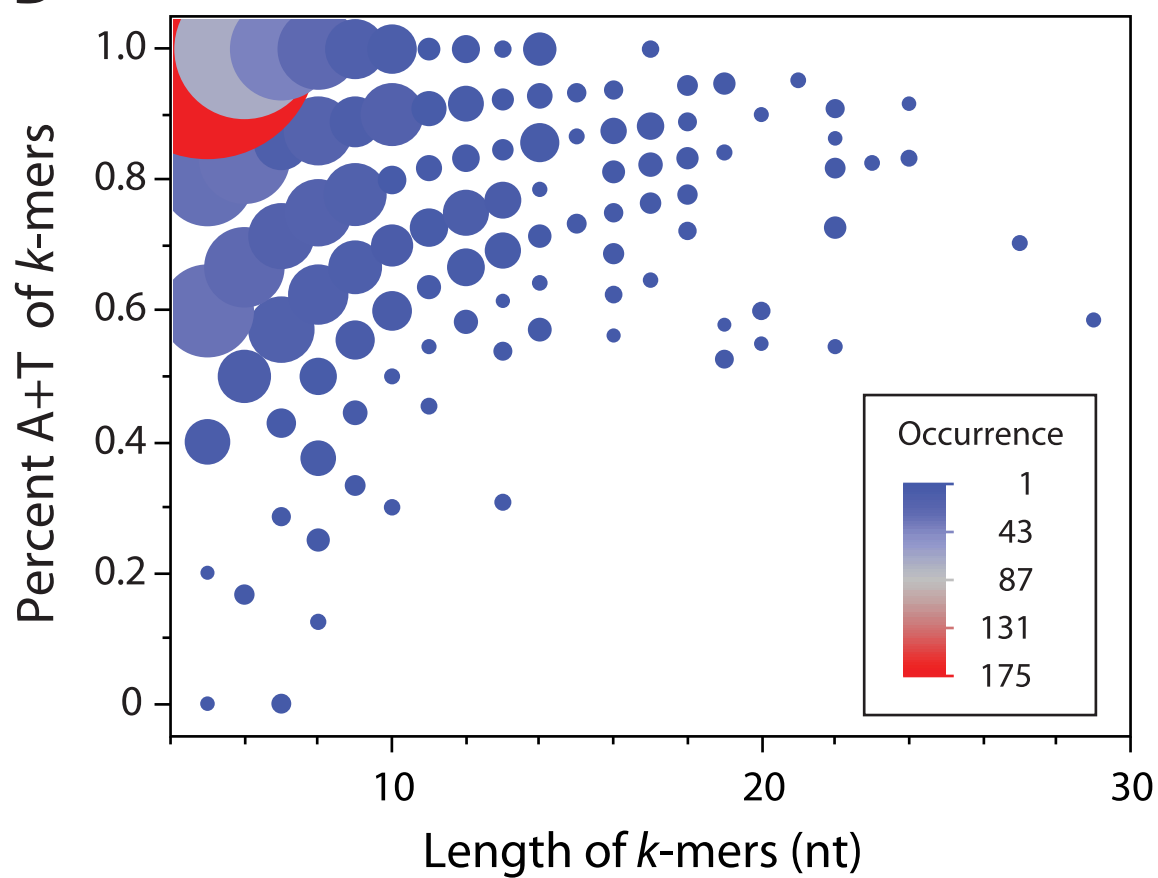

Supplement: Figure S3 — Evaluation of the base composition of conserved k-mers. (A) The distribution of base compositions for k-mers are compared to entire IGS from Buchnera-Ap and (B) compared by k-mer lengths. In (B) the size and color of spheres denote the relative occurrence of k-mers of a particular size and A+T content. (PDF) [file pgen.1002252.s003.pdf]
